# Machine Learning Models to Predict Primary Sites of Metastatic Cervical Carcinoma From Unknown Primary

**DOI:** 10.3389/fgene.2020.614823

**Published:** 2020-12-21

**Authors:** Di Lu, Jianjun Jiang, Xiguang Liu, He Wang, Siyang Feng, Xiaoshun Shi, Zhizhi Wang, Zhiming Chen, Xuebin Yan, Hua Wu, Kaican Cai

**Affiliations:** ^1^Department of Thoracic Surgery, Nanfang Hospital, Southern Medical University, Guangzhou, China; ^2^Department of Thoracic Surgery, Peking University Shenzhen Hospital, Shenzhen, China

**Keywords:** metastatic cervical carcinoma from unknown primary, random forest, neural network, support vector machine, predict, primary sites

## Abstract

Metastatic cervical carcinoma from unknown primary (MCCUP) accounts for 1–4% of all head and neck tumors, and identifying the primary site in MCCUP is challenging. The most common histopathological type of MCCUP is squamous cell carcinoma (SCC), and it remains difficult to identify the primary site pathologically. Therefore, it seems necessary and urgent to develop novel and effective methods to determine the primary site in MCCUP. In the present study, the RNA sequencing data of four types of SCC and Pan-Cancer from the cancer genome atlas (TCGA) were obtained. And after data pre-processing, their differentially expressed genes (DEGs) were identified, respectively. Gene ontology (GO) and Kyoto Encyclopedia of Genes and Genomes (KEGG) pathway analysis indicated that these significantly changed genes of four types of SCC share lots of similar molecular functions and histological features. Then three machine learning models, [Random Forest (RF), support vector machine (SVM), and neural network (NN)] which consisted of ten genes to distinguish these four types of SCC were developed. Among the three models with prediction tests, the RF model worked best in the external validation set, with an overall predictive accuracy of 88.2%, sensitivity of 88.71%, and specificity of 95.42%. The NN model is the second in efficacy, with an overall accuracy of 82.02%, sensitivity of 81.23%, and specificity of 93.04%. The SVM model is the last, with an overall accuracy of 76.69%, sensitivity of 74.81%, and specificity of 90.84%. The present analysis of similarities and differences among the four types of SCC, and novel models developments for distinguishing four types of SCC with informatics methods shed lights on precision MCCUP diagnosis in the future.

## Introduction

Metastatic cervical carcinoma from unknown primary (MCCUP) is defined as metastatic disease in the lymph nodes of the neck without any evidences of a primary tumor after appropriate investigations. It is a type of cancer which originates from unknown primary sites, and squamous cell histology is prominent, accounting for 75–90% of cases (Arosio et al., 2017; Jereczek-Fossa et al., 2004). The special features of the lymphatic drainage of the head and neck regions suggest that the primary sites may locate in head, neck (oropharynx, larynx, and tongue) or thorax (tracheal, bronchial, lung, and esophagus) (Jereczek-Fossa et al., 2004; Arosio et al., 2017). However, despite comprehensive diagnostic work-ups including fibroscopy, computed tomography, magnetic resonance imaging, positron emission tomography, fine-needle aspiration, and panendoscopy have been conducted, the primary site remains difficult to identify in cases of  MCCUP. An accurate identification of the primary site is crutial for the designment of effective treatments. Therefore, the developments of a novel and effective method to determine the primary site in MCCUP seem rather necessary and urgent.

The reasons for the failure of primary tumor diagnosis is not fully elaborated; however, the small size of the primary tumor may increase the difficulties in identifying the primary site and the evolutions of the cancer cell itself may be one of the reasons too (Arosio et al., 2017). The developments of high-throughput and next-generation sequencing technologies have improved our understanding of the molecular landscape of cancer, offering the basis and possibility to discover predictive biomarkers for cancer diagnosis (Roychowdhury and Chinnaiyan, 2016). Relevant high-throughput studies indicate that squamous cell carcinoma (SCC) shares certain common histological characteristics and molecular signatures (Dotto and Rustgi, 2016; Campbell et al., 2018) which makes it more difficult to identify the primary site of MCCUPwhose pathologic type is primarily SCC. In cases of MCCUP, determining the primary site is challenging.

Research discoveries derived through cancer genome and transcriptome studies have potential clinical impact as biomarkers (Roychowdhury and Chinnaiyan, 2016). And machine learning approaches have been applied to cancer prognosis and prediction and shown significant advantage in differential diagnosis (Cruz and Wishart, 2007). Khan et al. (2001) developed a model of Neural Networks (NN) for diagnostic classification base on gene-expression signatures of the small, round blue cell tumors (SRBCTs) of childhood, of which four subtypes share similar appearance on routine histology. Yang et al. (2018) used the Random Forest (RF) algorithm to select biomarker metabolites and establish a diagnostic model in a metabolomics study of cancer cachexia.

In the present study, a dataset from The Cancer Genome Atlas (TCGA) RNA-Seq data of squamous cancer and TCGA Pan-Cancer (PANCAN) data were employed to conduct a series of bioinformatics analyses, and three machine learning models [RF, NN, support vector machine (SVM)] were developed to explore the potantial effective tool to distinguish these squamous cancers.

## Materials and Methods

### Data Source and Data Pre-Processing

The Cancer Genome Atlas RNA-Seq data of four types of cancer [Genomic Data Commons (GDC) TCGA Cervical Cancer (CESC), GDC TCGA Esophageal Cancer (ESCA), GDC TCGA Head and Neck Cancer (HNSC), GDC TCGA Lung Squamous Cell Carcinoma (LUSC)], and the phenotype data and TCGA PANCAN data were downloaded from University of California Santa Cruz (UCSC) Xena database^1^. The GDC sample sheet of all squamous cell carcinomas of TCGA database^2^ were downloaded from TCGA database by using the searching strategy (Disease Type IS squamous cell neoplasms AND Program Name IS TCGA AND Experimental Strategy IS RNA-Seq).

Based on sample ID in the GDC sample sheet, the samples of SCC in the data PANCAN were extracted. Using the same methods we extracted the SCC data of the four types of cancer data (CESC, ESCA, HNSC, HNSC). Then we renamed above data as PANCANsqu, CESC, ESCC, HNSC, and LUSC spectively. Using the function Rtsne provided by R Rtsne to visualize PANCANsqu based on t-distributed stochastic neighbor embedding (t-SNE) algorithm (van der Maaten and Hinton, 2008; van der Maaten, 2014).

### Differential Expression Analysis

The DESeq2 R package was used to identify differentially expressed genes (DEGs) of each squamous cancer data (CESC, ESCC, HNSC, and LUSC) (Love et al., 2014, 2). Padjust < 0.01 and absolute log2 FC > 2 were chosen as the cut-off criteria. The Venn diagram was generated by VennDiagram R package.

Gene ontology (GO) and Kyoto Encyclopedia of Genes and Genomes (KEGG) pathway enrichment analysis

GO and KEGG pathway enrichment analysis was performed using clusterProfiler R package (Ashburner et al., 2000; Kanehisa and Goto, 2000; Yu et al., 2012). The enriched biological processes (BP), cellular components (CC), and molecular functions (MF) were obtained to analyze the DEGs of each cancer at the functional level. *P* < 0.01 was set as the threshold value.

### Protein-protein Interaction Network Construction

The STRING online database^3^ was used for analyzing the protein-protein interaction (PPI) of the DEGs of each cancer, and Cytoscape software^4^ was used to visualize the PPI network of the DEGs (Snel et al., 2000; Shannon et al., 2003; Szklarczyk et al., 2019).

### Predict Model Construction and Validation

The function nearZeroVar was used to identify and eliminate zero and near-zero-variance variables, and the function findCorrelation to remove Correlated variables with absolute correlations above 0.9, and the function findLinearCombos to eliminate the linear dependencies (Kuhn, 2008). The above three functions are provided by the R caret R package (Kuhn, 2008). Feature selection using recursive feature elimination algorithms (Guyon et al., 2002).

The function createDataPartition was used to create balanced splits of the PANCANsqu data, creating a single 70/30% split of the data. Then the 70% split of the data was used as training set while the remaining 30% data was used as the validation set. Several machine learning methodologies [RF, NN, and SVM] were adopted to construct the model with data of the training set using caret, e1071 and randomForest R package and 10 fold cross validation is applied in model training (Kuhn, 2008).

Sensitivity, specificity, and area under curve (AUC) values were determined to evaluate the performance of the established classifier in the validation set.

The modeling process is briefly described below:

Fristly, the function trainControl was used to define the parameters of sampling and cross-validation.

method = “repeatedcv”, number = 10, repeats = 3, returnResamp = “all”, classProbs = T

Secondly, The function train was used to build three training models.

1. method = “rf”, mtry = 2
2. method = “svmRadial”, sigma = 0.3469467 and C = 1
3. method = “nnet”, size = 5 and decay = 0.1

Lastly, The function predict was used to predict the sample type base on the training model, function extractPrediction and extractProb to acquire the model prediction results and their probabilities.

To evaluate the performance of the model that have been established, the function confusionMatrix was used to obtain the confusion matrix and the ROCR R package to draw the ROC curve.

## Results

### Expression Profiles of Four Types of Squamous Cancer

**FIGURE 1:**
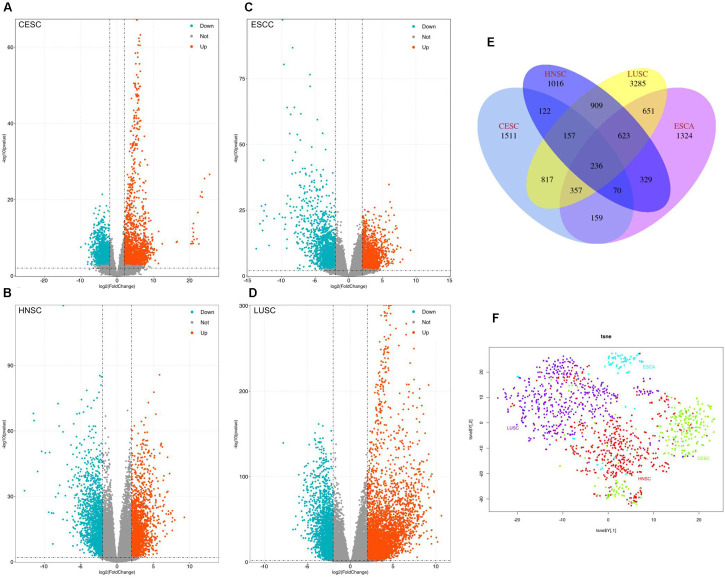
Identification of the differentially expressed genes (DEGs) between tumor tissues and normal controls, **(A–D)** Volcano plots of genes showing significantly different expression between tumor tissues and normal controls. The *Y*-axis indicates the *p-*values (log10 scaled), whereas the *X*-axis shows the fold change (log2 scaled). Each symbol represents a different gene, and the red/blue colors of the symbols categorize the upregulated/downregulated genes under different criteria (*p*-value and fold change threshold). *p-*value < 0.01 was considered statistically significant, whereas log2 (fold change) = 2 was set as the threshold. **(E)** Venn diagram of the DEGs of the four squamous carcinoma. **(F)**: Visualized PANCANsqu RNA-seq data used by t-SNE algorithm.

Volcano plots were generated to visualize the distribution of expressed genes between cancer and normal controls from the four RNA-Seq data (CESC, ESCC, HNSC, and LUSC). Red or green dots in the plots represent significantly upregulated or downregulated genesrespectively (Figures 1A–D). Venn diagrams show the DEGs information among CESC, ESCC, HNSC, and LUSC (Figure 1E). In total, 3429, 3749, 3462, and 7035 DEGs were identified from the four RNA-Seq data of ESCC (CESC, ESCC, HNSC, and LUSC). A total of 236 common DEGs were significantly changed in all four independent cohorts, and 1511(CESC), 1324(ESCC), 1016(HNSC), and 3285(LUSC) specific DEGs were identified in the difference set (just in one type of cancer). Detailed information of the DEGs is provided by Supplementary Materials.

### GO and KEGG Pathway Enrichment Analysis

**FIGURE 2:**
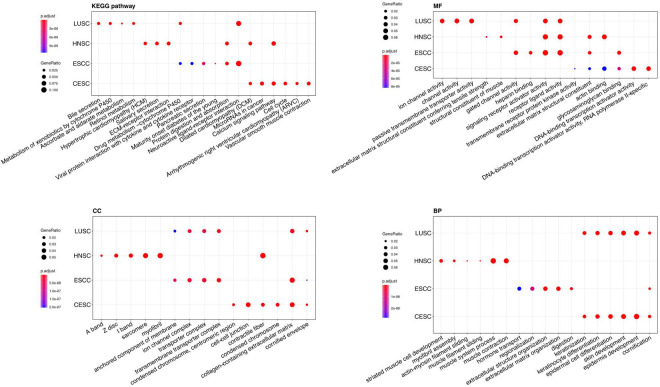
Gene Ontology (GO) analyses and Kyoto Encyclopedia of Genes and Genomes (KEGG) pathway analysis of DEGs of the four types of carcinoma (LUSC, HNSC, ESCC, and CESC). MF, molecular function; CC, cellular component; BP, biological process. The *Y*-axis represents the four types of carcinoma.

GO and KEGG pathway enrichment analyses of DEGs were performed using clusterProfiler R package, and the results were shown in the Figure 2.

For “BP”, CESC and LUSC both showed enrichment in cornification, epidermis development, skin development, epidermal cell differentiation, keratinocyte differentiation, and keratinization. The differential DEGs expressed in ESCC mainly showed enrichment in digestion, extracellular structure organization, extracellular matrix organization, regionalization and hormone transport. The DEGs expressed in HNSCC mainly showed enrichment in response to muscle contraction, muscle system process, muscle filament sliding, actin-myosin filament sliding, myofibril assembly and striated muscle cell development.

For the “cellular component (CC)” ontology, ESCC and LUSC both showed enrichment in transmembrane transporter complex, transporter complex, ion channel complex, anchored components of membrane. The differential genes expressed in HNSC mainly showed enrichment in myofibril contractile fiber part, sarcomere, I band and Z disc. The differential genes expressed in CESC showed enrichment in cornified envelope, collagen-containing extracellular matrix, condensed chromosome, contractile fiber and cell-cell junction.

Regarding “MF”, the DEGs of ESCC and HNSCC both showed enrichment in extracellular matrix structural constituent, receptor ligand activity and passive transmembrane transporter activity. The differential genes expressed in LUSC mainly showed enrichment in substrate-specific channel activity, ion channel activity, ion gated channel activity and gated channel activity. The differential genes expressed only in CESC mainly showed enrichment in DNA-binding transcription activator activity, RNA polymerase II-specific glycosaminoglycan binding, actin binding and extracellular matrix structural constituent.

For “KEGG pathway enrichment analysis”, LUSC mainly showed enrichment in Retinol metabolism, ascorbate and aldarate metabolism, Metabolism of xenobiotics by cytochrome P450 and Bile secretion. The differential DEGs expressed in ESCC mainly showed enrichment in Neuroactive ligand-receptor interaction, Protein digestion and absorption, Maturity onset diabetes of the young, Pancreatic secretion, Viral protein interaction with cytokine and cytokine receptor and Drug metabolism – cytochrome P450. The DEGs expressed in HNSCC mainly showed enrichment in Hypertrophic cardiomyopathy (HCM), ECM-receptor interaction, Salivary secretion, Calcium signaling pathway, and Dilated cardiomyopathy (DCM).

### Identification of Key Candidate Genes With the PPI Network of DEGs

**FIGURE 3:**
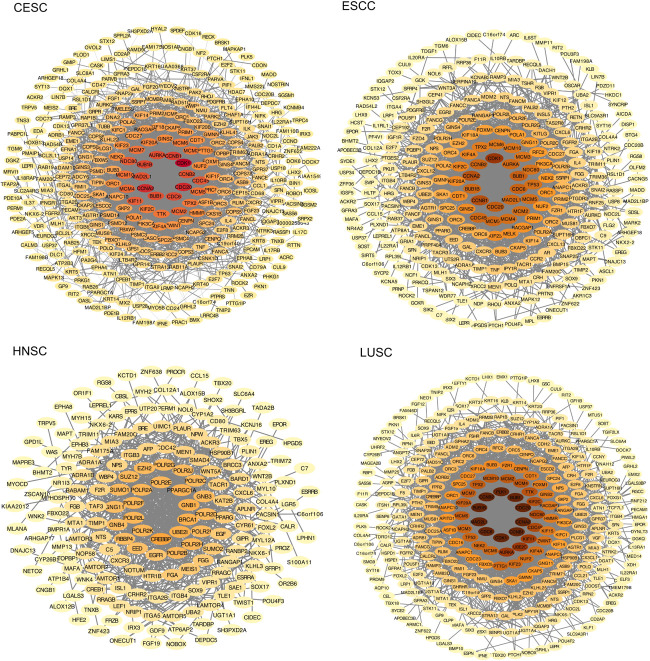
The protein-protein interaction (PPI) network of the differentially expressed genes (DEGs). The color of nodes represents the number of connections, the darker the color, the more connections.

The PPI network of the four types of squamous cancer was constructed using the STRING online database and Cytoscape (Figure 3). Then the central node genes (more than 10 connections/interactions) were identified. In the DEGs of CESC, the top ten highly connected genes were CDK1, CDC20, CCNA2, CCNB1, BUB1B, CDC6, BUB1, AURKA, CCNB2, and MAD2L1. In the DEGs of ESCC, the top ten highly connected genes were CDK1, CCNB1, CCNA2, CDC20, BUB1, CDC6, CCNB2, CDC45, MAD2L1, and BUB1B. In the DEGs of HNSC, the top ten highly connected genes were CREBBP, BRCA1, UBE2I, GNB1, PPARGC1A, POLR2F, POLR2A, POLR2H, POLR2B, and POLR2K. In the DEGs of LUSC, the top ten highly connected genes were CDK1, CCNB1, CCNA2, CDC20, BUB1, PLK1, CCNB2, BUB1B, MAD2L1, and CDC6.

### Model Construction for Discriminating the Four Types of Squamous Cancer

The t-SNE algorithm was used to visualize PANCANsqu data prior to model construction, as shown in Figure 1F. After preprocessing the PANCANsqu data, 1,327 variables were retained. To improve model predictive accuracy and reduce model complexity, we used recursive feature elimination as the methods of feature selection by using the function rfe in the caret R package. Then the number of the variables (5, 10, 15, 30, 60, 80, and 1,327) was tested in the feature selection procedure, and it was found that 80 genes worked the best with the accuracy of 93.35%. Table 1 showed the detail information. As the performance of each number of variable showed in Table 1, considering the model predictive accuracy and the model complexity, the top 10 gene were selected for the subsequent researches, which were C11orf85, LA16c-431H6.6, MYBPH, MAP9, FMO2, SCGB3A1, BPIFA1, TBX1, SRRM2, and AC016549.1.

**TABLE 1:** The performance of each different number of variable tested in the feature selection procedure by using the function rfe in caret R package.

Variables	Accuracy	Kappa	Accuracy SD	Kappa SD
5	0.8256	0.7401	0.01802	0.02615
10	0.9034	0.8565	0.02822	0.04214
15	0.9209	0.8823	0.01809	0.02695
30	0.9321	0.8988	0.01848	0.02770
60	0.9279	0.8926	0.02110	0.03159
80	0.9335	0.9009	0.02211	0.03303
1327	0.9125	0.8688	0.02950	0.04434

**FIGURE 4:**
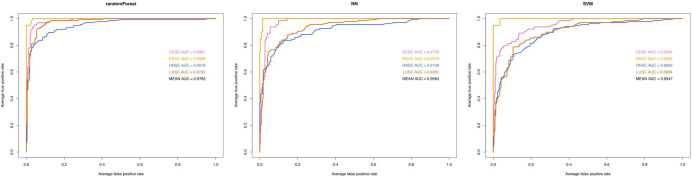
Predictive performance of three machine learning methodologies [Random Forest (RF), Neural Networks (NN), support vector machine (SVM)]. Receiver operating characteristic curve analysis of three machine learning model for classifying the four types of carcinoma (LUSC, HNSC, ESCC, and CESC). AUC, area under curve.

Several statistical methodologies (RF, SVM, and NN) were used to construct the prediction model with data from the training set, and for each statistical methodology, using subsets of DEGs (the top 10 genes generated by feature selection) instead of all DEGs as variables. Receiver operating characteristic curves were used to evaluate the predictive value (Figure 4). Among the three statistical methodologies tested, the RF model worked best both in the training set and in the external validation set (Table 2), with an overall predictive accuracy of 88.2%, mean sensitivity of 88.71%, and mean specificity of 95.42%. Mean AUC for the validation sets was 0.9782. Subsequently, for the NN model, overall predictive accuracy was 0.8202, mean sensitivity was 0.8123, mean specificity was 0.9304, and the mean AUC was 0.9563. For the SVM model, overall predictive accuracy was 0.7669, mean sensitivity was 0.7481, mean specificity was 0.9084, and the mean AUC was 0.9347.

**TABLE 2:** Statistical models for discriminating the four types of carcinoma (LUSC, HNSC, ESCC, and CESC) and their predictive performances.

Method	Accuracy	Sensitivity	Specificity	AUC
RF	0.882	0.8871	0.9542	0.9782
NN	0.8202	0.8123	0.9304	0.9563
SVM	0.7669	0.7481	0.9084	0.9347

*RF, random forest; NN, neural networks; SVM, support vector machine; AUC, area under curve.*

## Discussion

In this study, we investigated methods for the accurate diagnosis of the primary site of  MCCUP using microarrays of four potential primary tissues (CESC, ESCC, HNSC, and LUSC). We identified significant DEGs from four RNA-Seq data. The similarities and differences among the four types of squamous cancer were then analyzed using bioinformatics methods based on these significant DEG sets. Based on the data of TCGA PANCAN, a predictive RF model consisting of a ten-gene signature was established that could effectively discriminate between the four types of carcinoma.

The four potential primary sites for MCCUP, CESC, ESCC, HNSC, and LUSC shared some common features. GO and KEGG pathway enrichment analyses showed clear similarities and differences among these four types of carcinoma. The KEGG pathway in which the DEGs of these four cancers are enriched, respectively, is rarely the same. The PPI network analysis provided detailed interactions/connections among the common DEGs. In the PPI network of the four types of squamous cancer, the top ten highly connected genes were selected. It is clear that in the top ten genes in PPI network of CESC, ESCC, and LUSC, nine of them (BUB1, BUB1B, CCNA2, CCNB1, CCNB2, CDC20, CDC6, CDK1, and MAD2L1) are the same.

BUB1 mitotic checkpoint serine/threonine kinase (BUB1), BUB1 mitotic checkpoint serine/threonine kinase B (BUB1B), both of them play a central role in mitosis which are reported associated with aneuploidy and several forms of cancer (Siemeister et al., 2019). Cyclin A2(CCNA2), cyclin B1(CCNB1), and cyclin B2(CCNB2) are essential components of the cell cycle regulatory machinery. Several researches showed that CCNB2 overexpression was associated with poor prognosis in human hepatocellular carcinoma, non-small cell lung cancer patients and invasive breast carcinoma (Shubbar et al., 2013; Qian et al., 2015; Li et al., 2019). Cell division cycle 20 (CDC20) is a regulatory molecule that plays critical roles at multiple points of the cell cycle and may serve an oncogenic role in human cancer (Chu et al., 2019). A study showed that CDC20 contributed to the developments of human cutaneous SCC through the Wnt/β-catenin signaling pathway (Chu et al., 2019). Cell division cycle 6(CDC6) might be a biomarker of high grade and invasive lesions of the cervix which was reported previously (Murphy, 2005, 6). Cyclin dependent kinase 1 (CDK1) is essential both for cell division in the embryo and inhibition of CDK1 induces cell death in human tumor cells (Goga et al., 2007; Malumbres and Barbacid, 2009, 1). Mitotic arrest deficient 2 like 1 (MAD2L1) is the gene controlling mitosis whose expression was found to be involved in carcinogenesis and prognosis of small cell lung cancer (Wu et al., 2018, 2).

Research discoveries derived from cancer genome and transcriptome studies have potential clinical impacts on biomarkers (Roychowdhury and Chinnaiyan, 2016). Machine learning approaches have been applied to cancer prognosis and prediction (Cruz and Wishart, 2007). RF is one of machine learning algorithms used for supervised learning, which can be used for both classification and regression tasks too. The pros of Random Forests are that it is a relatively fast and powerful algorithm which can be parallelized and performs well on many problems, and even with small datasets, the output returns satisfying prediction probabilities. Zhou et al. (2017) used the RF classifier to select feature genes from mRNA microarray data to diagnose renal fibrosis. Han et al. (2019) used RF to predict the developments of end-stage renal diseases in immunoglobulin nephropathy patients. SVM is a novel machine learning method that simplifies the usual classification and regression problems. A small number of support vectors determine the final results and are not sensitive to outliers. This helps us eliminate large number of redundant samples and grasp key samples, which makes us avoid the sense of “dimensionality disaster” and enables the algorithm to have good “robustness.” The SVM classifier is well suitable for signature modeling (Fan et al., 2004). Guyon et al. (2002) used the SVM classifier to select feature genes from DNA microarrays and showed great classification performances. Fan et al. (2004) proved that the SVM classifier used for feature gene selection could speed up the classification process and generalization performances. NN is a parallel computing model to the human neural structures, which has basic characteristics such as learning, memory, and inductions of the human brain and can process continuously, discrete data and predict data. Besides, it has strong robustness, memory ability, non-linear mapping ability and strong self-learning ability. Selvaraj et al. (2018) used NN algorithms to identify candidate drugs in a lung adenocarcinoma research. Shaabanpour Aghamaleki et al. (2019) applied the NN in order to identify a molecular biomarker for rapid leukemia diagnosis from blood samples and evaluate its potential for the detection of cancer.

However, there are no studies using machine learning approaches for the diagnosis of MCCUP. In the present study, three statistical methodologies were used to construct a prediction model using data from the training set. For each statistical methodology, the use of subsets of DEGs instead of all DEGs improved the predictive performance. Among the three statistical methodologies (RF, NN, and SVM) used to construct the prediction model, the ten gene RF model including C11orf85, LA16c-431H6.6, MYBPH, MAP9, FMO2, SCGB3A1, BPIFA1, TBX1, SRRM2, and AC016549.1 showed the best performance both in the training set and in the external validation set.

The ten-gene signature capability of effectively differentiating the four types of squamous carcinoma has potential diagnostic value in MCCUP. The training set and validation cohorts were retrospective, therefore these findings must be validated prospectively in future studies. In addition, we just analyzed four potential primary sites of MCCUP, future studies should include additional potential primary sites of  MCCUP and more extensive data, as well as more complex machine learning methods.

In conclusion, the present study analyzed the similarities and differences among CESC, ESCC, HNSC, and LUSC, which are four potential primary sites of MCCUP. A ten-gene predictive RF model was established based on the RNA-Seq data of the four types of carcinoma, which might have clinical utility for the accurate diagnosis of MCCUP and provide useful guidance for personalized and precision therapy.

## Data Availability Statement

The datasets for this study can be found in The Cancer Genome Atlas (TCGA) and the University of California Xena (UCSC Xena), Datasets link: https://portal.gdc.cancer.gov/repository and https://xenabrowser.net/datapages/.

## Author Contributions

DL and KC designed the study. DL, JJ, and XL were primarily responsible for conceptualization, methodology, and writing – reviewing and editing and contributed to this study equally. JJ and XL were responsible for data curation, software, and writing – original draft preparation. HW, SF, XS, and ZW were responsible for data revision. ZC, XY, HW, and KC revised the manuscript. All authors contributed to the article and approved the submitted version.

## Conflict of Interest

The authors declare that the research was conducted in the absence of any commercial or financial relationships that could be construed as a potential conflict of interest.

## Footnotes

1 https://xenabrowser.net/datapages/

2 https://portal.gdc.cancer.gov/

3 http://string-db.org

4 http://www.cytoscape.org/
